# Semi-quantitative evaluation of signal intensity and contrast-enhancement in Modic changes

**DOI:** 10.1186/s41747-017-0009-2

**Published:** 2017-06-29

**Authors:** Marta Tibiletti, Cristina Ciavarro, Vlasta Bari, Iain W. McCall, Jill P. G. Urban, Marco Brayda-Bruno, Fabio Galbusera

**Affiliations:** 1 Department of Internal Medicine II—Cardiology, Ulm University, Helmholtzstrasse 16, 89081 Ulm, Germany; 2 IRCCS Galeazzi Orthopedic Institute, Via Galeazzi 4, 20161 Milan, Italy; 3 IRCCS Policlinico San Donato, Piazza Edmondo Malan 1, 20097 San Donato Milanese, Italy; 4 Keele University, Keele, ST5 5BG UK; 5 Department of Physiology, Anatomy and Genetics, Oxford University, Oxford, OX1 3PA UK; 6 Department of Spine Surgery III, IRCCS Galeazzi Orthopaedic Institute, Via Galeazzi 4, 20161 Milan, Italy

**Keywords:** Modic changes, Gadolinium-based contrast agent, Contrast enhancement, Magnetic resonance imaging, Spine

## Abstract

**Background:**

Semi-quantitative evaluation of Modic changes (MCs) has recently been proposed as a way to standardise and increase repeatability of clinical studies. This study is aimed at developing semi-quantitative measures of enhancement, given by contrast agent injection, on T1-weighted images in MCs, and to investigate their reliability and relation with MC types.

**Methods:**

Thirty-seven subjects suffering from low back pain underwent T1-weighted and T2-weighted turbo spin-echo sequences. Five minutes after the injection of a paramagnetic contrast agent, a second T1-weighted sequence was acquired. Regions of interest (ROIs) corresponding to MCs were selected manually on the unenhanced image; control ROIs in the “healthy” bone marrow were selected. For each ROI, the mean signal intensity (SI) of unenhanced pixels and the mean absolute and normalised difference in SI between unenhanced and contrast-enhanced pixels values were calculated.

**Results:**

A total of 103 MCs were recognised and 61 were semi-quantitatively analysed: 16 type I, 34 type II and 11 type I/II. Regarding controls, MCs I showed a lower SI on the unenhanced T1-weighted images and a marked contrast enhancement (CE); MCs II showed a higher SI than controls on unenhanced images and a lower or comparable CE; and MCs I/II presented an intermediate SI on the unenhanced images and a marked CE. Inter-rater and intra-rater agreements were found to be excellent or substantial.

**Conclusions:**

Semi-quantitative measurements could differentiate MC types in terms of unenhanced SI and of CE with respect to “healthy” bone marrow.

## Key points

- Contrast enhancement differed between Modic changes and healthy bone marrow.
- Type I Modic changes showed a marked contrast enhancement.
- Type II Modic changes showed a low contrast enhancement.

## Introduction

Modic changes (MCs) are common variations of signal intensity in the endplate and vertebral body seen on magnetic resonance imaging (MRI) [1, 2]. Depending on their characteristics on T1-weighted and T2-weighted images, MCs are classified into type I (hypointense on T1 images and hyperintense on T2 images), type II (hyperintense on both T1-weighted and T2-weighted images) and type III (hypointense in both T1-weighted and T2-weighted images). A normal endplate is considered grade 0, and mixed types can co-exist in the same endplate [3]. MC types can change with time, but no fixed evolution pattern has been recognised [4]. MCs I are thought to represent an ongoing active degenerative process demonstrated by fissured endplates with adjacent vascular granulation tissue within the bone marrow [1]. MCs II are considered signs of fatty marrow degeneration [1]: fatty marrow, which consists mainly of fat cells, is characterised by sparse vascularisation [5]. MCs I commonly progress to MCs II, and mixed-type MCs I/II are observed frequently [3, 6]. Although MCs II are believed to be more stable [1], transition to MCs I has also been documented [7].

The relationship between low back pain (LBP) and MCs is controversial, although it has been suggested that MCs could help to classify patients suffering from non-specific LBP [8] and could influence the outcome of surgical treatment [9–12]. Conflicting results have been obtained in different studies, possibly in part due to variations in study methodology and MC diagnosis. In most MC studies to date, evaluations are made with the naked eye and are thus subjective [13]. A semi-quantitative evaluation of MCs may be helpful in investigating the relationship between MCs, clinical signs of LBP and surgical outcome.

Although MCs are defined as signal intensity abnormalities, only two studies have recently proposed semi-quantitative analysis of their signal intensity [13, 14]. Wang et al. [13] in particular proposed semi-quantitative measures on T1-weighted and T2-weighted images and showed the reliability of such methods.

In the case of suspected tumours, infections and vascular malformations [15], spinal MRI examinations are often accompanied by the intravenous injection of a contrast agent, a gadolinium chelate; this is not the case for LBP, for which a contrast injection is not commonly executed. Because the contrast agent shortens the T1 relaxation time, the tissues where the contrast agent pools (typically vessels, hyperaemic tissues and joint spaces) result in a higher signal on contrast-enhanced T1-weighted images compared with unenhanced T1-weighted images [15], in proportion to the concentration of the contrast agent [16].

Given the different vascular characteristics of MCs, the aim of the present study was to evaluate the contrast enhancement of MCs on T1-weighted images, hypothesising differences among the different types of MCs, by means of a semi-quantitative analysis.

## Methods

### Population

Thirty-seven patients affected by LBP were enrolled prospectively in this study, in which the primary outcome was to study contrast diffusion in intervertebral discs [17]. The selected population included male and female subjects, with an age of 42.5 ± 9.1 years (mean ± standard deviation) and an age range from 18 to 60 years. Each patient received detailed information regarding the study protocol and gave her/his consent. Exclusion criteria were: age under 18 or over 60 years, contrast agent allergy, reduced renal function, and contraindications to MRI. The study was approved by the local ethical committee.

### Radiological evaluation

MRI of the lumbar spine was performed with a 1.5-T scanner (Avanto; Siemens, Erlangen, Germany) with a phased-array back coil. Standard examinations included routine sagittal and axial T1-weighted (repetition time = 500 ms, echo time = 13 ms) and T2-weighted (repetition time = 4180 ms, echo time = 104 ms) turbo spin-echo sequences as well as axial T2-weighted sequences. In addition, ProHance^®^ (gadoteridol; Bracco Diagnostics, Princeton, RI, USA), a paramagnetic macrocyclic non-ionic contrast agent, was injected at a dose of 0.2 mmol/kg and a second T1-weighted image was taken approximately 5 minutes after contrast injection. A higher dose with respect to standard clinical applications was used following the indications of a previous study about diffusion in the intervertebral disc [18].

A musculoskeletal radiologist with more than 30 years of experience noted the presence and the type of MCs in the endplates from T12–L1 to L5–S1. Only the MCs with a vertical height of more than 5 mm were considered for the semi-quantitative evaluation, but the presence of smaller MCs was noted [13].

### Semi-quantitative measure of Modic changes

From the scans showing at least one MC, the unenhanced slice where each MC had the greatest depth was selected; the same slice was selected for the contrast-enhanced series. Unenhanced and contrast-enhanced T1-weighted scans were co-registered to ensure alignment using Elastix, a registration toolkit based on the National Library of Medicine Insight Segmentation and Registration Toolkit (ITK) [19]. To this aim, two-dimensional affine registrations (six degrees of freedom) were performed.

**Fig. 1:**
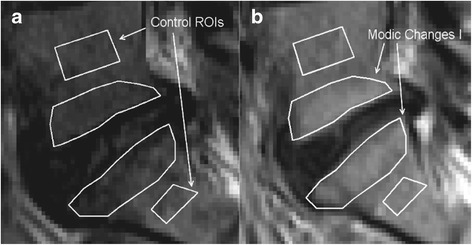
Example of ROI selection for MCs I and same vertebra control areas on (**a**) unenhanced and (**b**) contrast-enhanced images. Two MCs I are present at the lower endplate of L5 and the upper endplate of S1. *ROI* region of interest

**Fig. 2:**
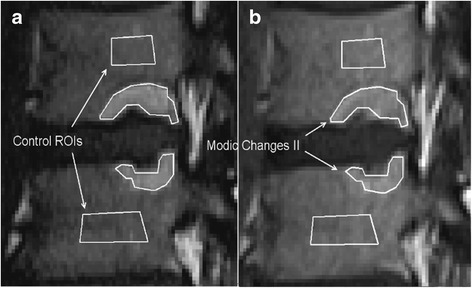
Example of ROI selection for MCs II and same vertebra control areas on (**a**) unenhanced and (**b**) contrast-enhanced images. MCs II are present at the lower endplate of L1 and the upper endplate of L2, surrounding an endplate defect. *ROI* region of interest

In order to obtain semi-quantitative data, software allowing for manual selection of a polygonal region of interest (ROI) on the unenhanced image (Figs. 1 and 2) and pixel-based calculation of the signal intensity of the selected area was developed in Matlab^®^ (MathWorks, Natick, MA, USA).

For each MC under consideration, the developed protocol asked the operator to select a ROI corresponding to the zone of altered intensity on the unenhanced image and two ROIs as controls, because a reference for the “healthy” bone marrow in at least one control ROI was necessary. The ideal control ROI would be close enough to the MC to minimise the influence of local field fluctuation [20], but also wide enough and free of signal alteration, which in some cases would be possible only in another site. These considerations lead to an investigation of two different kinds of control ROI:

1. a ROI in the same vertebra affected with the MC (same vertebra [SV]);
2. a ROI corresponding to the section of the closest upper vertebra without MC—in this case one ROI was used as reference for all the MCs in the same image (other vertebra [OV]).

For each ROI, the following three indexes were calculated:

1. mean value of the pixels encompassed in the ROI (PRE):
  $$ P R E= mean\left( RO{I}_{pre}\right) $$
2. mean value of the difference between post and pre contrast signal intensity (DIFF):
  $$ \mathrm{DIFF} = \mathrm{mean}\ \left({\mathrm{ROI}}_{\mathrm{post}}-{\mathrm{ROI}}_{\mathrm{pre}}\right) $$
3. ratio between PRE and DIFF, multiplied by 100 (NORM.DIFF):
  $$ NORM. DIFF=\left(\frac{DIFF}{PRE}\right)*100 $$

The three indexes calculated per ROI encompassing an MC were normalised with respect to the relevant control ROIs, to calculate the normalised signal intensity (NSI), as follows:

$$ {\mathrm{NSI}}_{\mathrm{PRE}}=\frac{\left({\mathrm{PRE}}_{\mathrm{MC}}-{\mathrm{PRE}}_{\mathrm{CONTROL}}\right)}{{\mathrm{PRE}}_{\mathrm{CONTROL}}}*100 $$

NSI was extracted for PRE, DIFF and NORM.DIFF values and for each control ROI (SV, OV), for a total of six indexes.

In order to analyse inter-rater and intra-rater reliability, this procedure was repeated by the same operator 5 months after the first evaluation. A second operator, a resident in radiology with 3 years of experience not directly involved in the research, received a brief explanation about the software and the aim of the study before rating all data in one session.

### Statistical analysis

Inter-rater and intra-rater agreement was analysed with the interclass correlation coefficient (ICC) (two-way mixed model, type absolute agreement), taking into consideration that an ICC of 0–0.2 represents slight agreement, 0.21–0.4 fair agreement, 0.41–0.6 moderate agreement, 0.61–0.8 substantial agreement and 0.81–1 excellent agreement [21].

The existence of significant differences in the described indexes among MC I, MC II and MC I/II was evaluated with a rank-sum test or Kruskal–Wallis one-way analysis of variance. The choice of a non-parametric test was justified by the non-normal distribution of data as confirmed by the Shapiro–Wilk test. If a statistical difference among groups was found, a multiple comparison procedure with Dunn’s method was performed to establish the existence of difference among pairs. One-sample *t* tests were carried out to test whether data had a mean significantly different with respect to zero. The presence of correlation between data was studied by Spearman rank-order correlation. Differences were considered significant when *p* < 0.050.

## Results

Among the 37 subjects enrolled in the study, 29 (78%) were diagnosed as having at least one MC. Of the 444 considered endplates, 103 (23%) had an MC and were classified as follows: 26 type I, 64 type II and 13 type I/II. Sixty-one MCs with a height greater than 5 mm were then considered for the semi-quantitative analysis: 16 type I, 34 type II and 11 type I/II. MCs type III were absent in all patients.

### Intra-rater and inter-rater agreement

Intra-rater agreement was excellent for all indexes considered (ICC 0.846–0.928). Inter-rater agreement was substantial or excellent (ICC 0.652–0.833). The type of control ROI did not influence reliability in any particular way.

### Control ROIs

The choice of control ROI had no significant effect on the calculated indexes when all MCs were considered together (NSI_PRE_, *p* = 0.715; NSI_DIFF_, *p* = 0.539; NSI_NORM.DIFF_, *p* = 0.971; rank-sum test). The same index calculated with respect to different control ROIs also showed high correlations: *R* ^2^ values calculated with Spearman correlation were 0.849 for NSI_PRE_, 0.79 for NSI_DIFF_ and 0.72 for NSI_NORM.DIFF_ (*p* < 0.001 for all).

### Index for different MC types

**Fig. 3:**
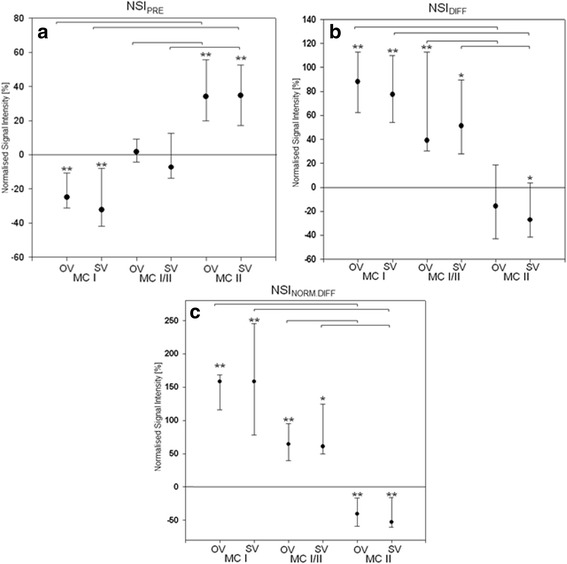
Median and 25th and 75th percentiles of NSI_PRE_ (**a**), NSI_DIFF_ (**b**) and NSI_NORM.DIFF_ (**c**) for MC type and control ROI in the other vertebra (*OV*) or in the same vertebra (*SV*). *p < 0.050, **p < 0.001 indicate whether the population has a median value significantly different with respect to zero. *Bars* indicate which pairs of MC type are significantly different (Dunn’s method, *p* < 0.050). NSI_PRE_ is higher than zero (*p* < 0.001) for MCs II, is lower than zero (*p* < 0.001) for MCs I and is not statistically different from zero for MCs I/II. MCs I and MCs I/II have a higher NSI_DIFF_ than MCs II, and MCs I and MCs I/II have a median NSI_DIFF_ higher than zero (all cases, *p* < 0.001), while for MCs II only for SV control was the median value lower than zero (*p* = 0.029; for OV, *p* = 0.213). MCs I and MCs I/II have a higher NSI_NORM.DIFF_ than MCs II; median NSI_NORM.DIFF_ for MCs I/II is lower than MCs I but is not statistically significant. NSI_NORM.DIFF_ median values for MCs I and MCs I/II are higher than zero (*p* < 0.001; but NSI_NORM.DIFF_ SV, *p* = 0.004), while MCs II have median values lower than zero (*p* < 0.001). *DIFF* mean difference between post and pre contrast signal intensity, *MC* Modic change, *NORM.DIFF* ratio between PRE and DIFF (×100), *NSI* normalised signal intensity, *PRE* mean pixels encompassed in the ROI

Figure 3 summarises the results of NSI_PRE,_ NSI_DIFF_ and NSI_NORM.DIFF_, per MC type and control ROI. Every index reported a strong statistical difference (*p* < 0.001) among MC types and was able to discriminate between MC I and MC II. However, no index was able to discriminate between MCs I and MCs I/II. In particular, NSI_PRE_ was higher than zero (*p* < 0.001) for MCs II, but lower than zero for MCs I (*p* < 0.001). For MCs I/II, NSI_PRE_ was not statistically different from zero. MCs I and MCs I/II had a higher NSI_DIFF_ than MCs II, and MCs I and MCs I/II had a median NSI_DIFF_ higher than zero (all cases, *p* < 0.001), while for MCs II only for SV control was the median value lower than zero (*p* = 0.029; for OV, *p* = 0.213). MCs I and MCs I/II had a higher NSI_NORM.DIFF_ than MCs II; the median NSI_NORM.DIFF_ for MCs I/II was lower than MCs I but the difference was not statistically significant. NSI_NORM.DIFF_ for MCs I and MCs I/II were higher than zero (*p* < 0.001; but NSI_NORM.DIFF_ SV, *p* = 0.004), while MCs II had median values lower than zero (*p* < 0.001).

**Fig. 4:**
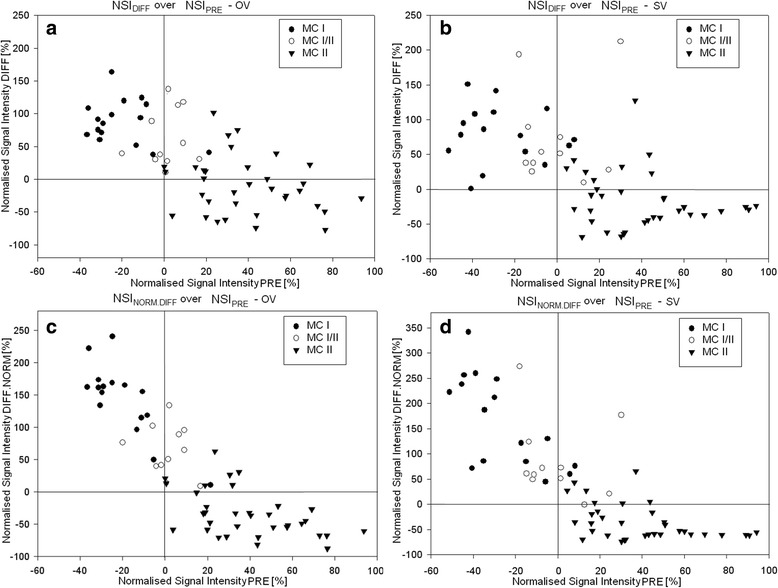
Scatter diagrams of NSI_DIFF_ over NSI_PRE_: **a** control areas in other vertebra (*OV*) and **b** control areas in the vertebra (*SV*). Scatter diagrams of NSI_NORM.DIFF_ over NSI_PRE:_ **c** control in OV, **d** control areas in SV. *Filled dots* MCs I, *empty dots* MCs I/II, *filled triangles* MCs II. MCs I pool on the *left* because their PRE values are mostly negative, while MCs II pool on the *right*, always being positive values. MCs I/II report NSI_PRE_ values around zero, with values ranging between –19.9 and +16.7% for OV and between –18 and +30.1% for SV. *DIFF* mean difference between post and pre contrast signal intensity, *MC* Modic change, *NORM.DIFF* ratio between PRE and DIFF (×100), *NSI* normalised signal intensity, *PRE* mean pixels encompassed in the ROI

Fig. 4 shows scatter plots of NSI_DIFF_ against NSI_PRE_ and of NSI_NORM.DIFF_ against NSI_PRE_ for each control ROI. MCs I pool on the left of each graphic, because their PRE values are mostly negative, while MCs II pool on the right, with positive values. Considering enhancement indexes for NSI_DIFF_ and NSI_NORM.DIFF_, MCs I were characterised by positive values, while MCs II presented mostly negative values for NSI_NORM.DIFF_ (7 of 34 for SV and for OV were higher than zero) and were more scattered for NSI_DIFF_ (9 ROIs report values higher than zero in SV, 14 for OV). MCs I/II always reported positive enhancement (apart from one case in NSI_NORM.DIFF_ SV, which is –1%).

## Discussion

In this study we developed semi-quantitative indexes to analyse contrast enhancement of MCs with respect to “healthy” bone marrow on T1-weighted images. MCs I consistently showed a lower signal intensity compared with controls on unenhanced T1-weighted images as expected, and a marked enhancement on contrast-enhanced T1-weighted images, significantly greater than the enhancement present in the “healthy” marrow. Conversely, MCs II showed a higher signal intensity on unenhanced T1-weighted images and a decreased or comparable enhancement with respect to controls. MCs I/II presented a behaviour generally intermediate between MCs I and MCs II, with a signal intensity on unenhanced T1-weighted images no different compared with the healthy marrow because higher and lower intensity zones tend to compensate for each other, and an enhancement close to MC I (see Figs. 3 and 4).

Considering the contrast enhancement as an indication of the grade of tissue vascularisation, these results are consistent with the suggestion that MCs I represent an inflammatory condition characterised by oedema and an augmented presence of small capillaries, while MCs II represent the substitution of haematopoietic marrow with fatty marrow, which possesses a sparse vascularity [1, 3].

We found that intra-rater agreement for the indexes considered was excellent, despite a time interval of 5 months between the two evaluations. Inter-rater agreement ranged between substantial and excellent, despite only providing a brief explanatory session to the resident before rating. These results are in agreement with the data on reliability on semi-quantitative measure of MC recently published by Wang et al. [13].

Wang et al. [13] speculated that the signal intensity of MCs on unenhanced T1-weighted images may indicate the severity of the degeneration. We verified that the contrast enhancement may add useful information about the degree of vascularisation in MCs, which strongly varies among types.

Moreover, contrast enhancement may help in the classification of mixed-type I/II MCs, which is more difficult and requires more skills than the classification of pure type I or type II MCs [22]. The mixed type may represent an intermediate stage in a process of conversion between pure categories of MCs [3], which may be connected with the progression of the pathologic process [23]. Thus, a reliable classification based on contrast enhancement may help in the study of temporal evolution of MCs and of the degenerative disorder during patient follow-up, and eventually help in the clarification of this process, whose origin and dynamics are yet to be completely understood.

We have evaluated the simple and normalised difference between unenhanced and contrast-enhanced images. The pure, non-normalised difference demonstrated a change in the contrast enhancement in different MCs with respect to controls. The normalised difference has the advantage of being able to incorporate information about unenhanced signal intensity and the contrast enhancement, and can thus be used as a reference measure. Normalisation tends to give more sparse results than difference, particularly for MCs I, due to division of low values.

Given the lack of reliable reference for T1-weighted images, it was necessary to identify a ROI unaffected by MC as a control. If possible, we selected a seemingly “healthy” area in the same vertebral body affected by the degeneration, thus minimising magnetic field alterations [19]. Alternatively, in the case of an extensive MC involving most of the vertebral body, we selected an area in the vertebral body of the upper vertebra not affected with MC. We did not observe a significant difference in the indexes calculated with different control ROIs and also found a high correlation between indexes extracted with different controls, suggesting that the choice of the control ROI only has a marginal influence on the results.

The first limitation of this study is the low number of MCs considered, due to the relatively low number of subjects enrolled. Other studies based on a wider population would be necessary to verify the possibility of establishing reference values of contrast enhancement for MCs, to investigate whether the indexes described may be an indicator of the severity grade of the degenerative process as indicated by clinical indexes. Indeed, besides contributing to the basic knowledge about contrast-enhanced MRI of the spine, the availability of a semi-quantitative index could be useful to evaluate the progression of the degenerative disorder. Given the risk associated with the injection of gadolinium chelates [24, 25], we recognise that the simple investigation of MCs cannot be considered a justification for a contrast-enhanced MRI of the spine. Nevertheless, because this examination is performed for specific purposes in patients with previous history of spine surgeries or for other conditions such as fever, immunosuppression or oncological history, data on contrast enhancement of MCs may be available for a non-negligible number of patients. Second, we included only MCs with a height greater than 5 mm, although an investigation of smaller MCs would be valuable for understanding the initiation mechanisms of endplate defects. This choice was related to the low spatial resolution of the images and the use of a subjective, manual method for the ROI creation, preventing an accurate and repeatable selection of the few pixels with altered signal intensity of small MCs. Third, T2-weighted fat-saturated images, commonly used to assess oedematous changes in MCs I [26], were not acquired in this study. Fourth, we did not investigate the possible correlation between MC enhancement and clinical data such as pain or quality of life scores, which were not collected in the primary study [17]. Finally, the study was conducted on a single 1.5-T scanner, and the findings are therefore to be considered valid only at that field strength.

In conclusion, based on the present results we do not recommend contrast injection for the MRI assessment of MCs alone, because they can be diagnosed qualitatively without contrast injection. However, the novel data reported interestingly show the difference in signal intensity of MCs on unenhanced and contrast-enhanced T1-weighted images with respect to “healthy” bone marrow: MCs I consistently showed lower signal intensity than control areas on unenhanced images and marked contrast enhancement; MCs II showed a higher signal intensity on unenhanced images and a decreased or comparable contrast enhancement when compared with control areas; and MCs I/II presented a behaviour generally intermediate between MCs I and MCs II (unenhanced signal intensity close to MCs II and contrast enhancement close to MCs I).
